# The causal integration ladder: a multilevel evidence framework for therapeutic target evaluation in cervical cancer

**DOI:** 10.3389/fsysb.2026.1874355

**Published:** 2026-09-18

**Authors:** Shishir Singh, Manyata Srivastava, Pragathi Uppada, Diksha Ameta, Aesha Singh, Monisha Banerjee, Atar Singh Kushwah

**Affiliations:** 1 Division of Immunobiology, Cincinnati Children’s Hospital Medical Center, Cincinnati, OH, United States; 2 Department of Biosciences, Manipal University Jaipur, Jaipur, Rajasthan, India; 3 Department of Internal Medicine, Marshall University, Huntington, WV, United States; 4 Department of Zoology,University of Lucknow, Lucknow, India

**Keywords:** causal inference, causal integration ladder, cervical cancer, evidence integration, functional genomics, human papillomavirus, therapeutic-target evaluation

## Abstract

Cervical-cancer genomic studies nominate many altered genes, but the evidence needed to distinguish disease association from a causal, therapeutically tractable mechanism is rarely stated explicitly. We present the Causal Integration Ladder (CIL), an evidence-accounting framework that distinguishes Level I association, inherited Level II-G evidence, acquired Level II-S evidence, Level III computational and direct functional evidence, and Level IV translational validation, while treating viral etiology as an explicit context modifier. An exploratory TCGA-CESC screen (306 tumours, 3 normal samples) supplied 25 Level I candidates. HPV annotations were available for 291 primary tumours (280 positive, 9 negative, 2 indeterminate). Restriction to HPV-positive tumours preserved the direction of all 25 Level I effects; HPV-positive versus HPV-negative comparisons were exploratory because the negative group was small and histologically heterogeneous. Somatic analysis used 194 mutation-evaluable and 295 copy-number-evaluable tumours. Ten candidates showed false-discovery-rate-significant copy-number–expression associations, including CDKN2A, whereas recurrent protein-altering mutation was uncommon. Eight genes were additionally audited using public cis-eQTL, GWAS, dependency, pharmacogenomic, and cell-compartment resources. The revised CIL reports inherited, somatic, etiological, and functional evidence independently; absence of germline support is not interpreted as evidence against somatic, viral, or functional relevance. No observational result is presented as experimental validation, and Level III direct perturbation and Level IV translational claims remain prospective.

## 1 Introduction

Persistent high-risk human papillomavirus (HPV) infection is the necessary etiological event for most cervical cancers, but viral oncogenesis is expressed through heterogeneous host-cell, immune, and stromal programs (McLaughlin-Drubin and Münger, 2009; Moody and Laimins, 2010; Kushwah et al., 2024). Transcriptomic and multi-omic studies can catalogue these programs at scale. They do not, by themselves, show whether a dysregulated gene drives the disease, marks an altered cell compartment, responds to tumour development, or is suitable for therapeutic intervention.

This distinction matters because statistical association, causal relevance, and targetability are different claims. Differential expression may reflect reverse causation or cell-composition shifts; a genome-wide association study (GWAS) signal may tag another causal locus; an expression quantitative trait locus (eQTL) may operate in a proxy tissue; and a dependency observed in a cell line may reflect general essentiality rather than a cancer-selective vulnerability (Richmond et al., 2016; Pingault et al., 2018; Porcu et al., 2021; Arafeh et al., 2025). Combining data modalities is useful only when the contribution and limitation of each modality are made explicit. Cervical-cancer studies of MGMT illustrate this distinction: genetic and epigenetic alterations may correlate with chemoradiotherapy response, but such associations still require mechanistic and treatment-context validation before they support causal or therapeutic claims (Gupta et al., 2023; Singh et al., 2025).

Genetically anchored approaches can narrow this gap. Colocalization evaluates whether molecular and disease associations are compatible with a shared causal variant, while Mendelian randomization (MR) estimates the direction and magnitude of an exposure–outcome relationship under instrument-validity assumptions (Giambartolomei et al., 2014; Davey Smith and Hemani, 2014). Functional screens provide an orthogonal layer by testing whether perturbation changes cellular fitness, but computational dependency and pharmacogenomic associations remain context dependent (Tsherniak et al., 2017; Behan et al., 2019). No individual method is sufficient.

We therefore propose the Causal Integration Ladder (CIL) as an evidence-accounting framework rather than a new statistical estimator. CIL records what type of claim is supported, what evidence is missing, and which study-quality modifiers limit confidence. It distinguishes genetic-evidence completeness at Level II (Full versus Partial) from computational functional strength at Level III-A (Strong versus Moderate) and explicitly reserves Level III-B and Level IV for direct experimental and translational validation (Figure 1).

**FIGURE 1:**
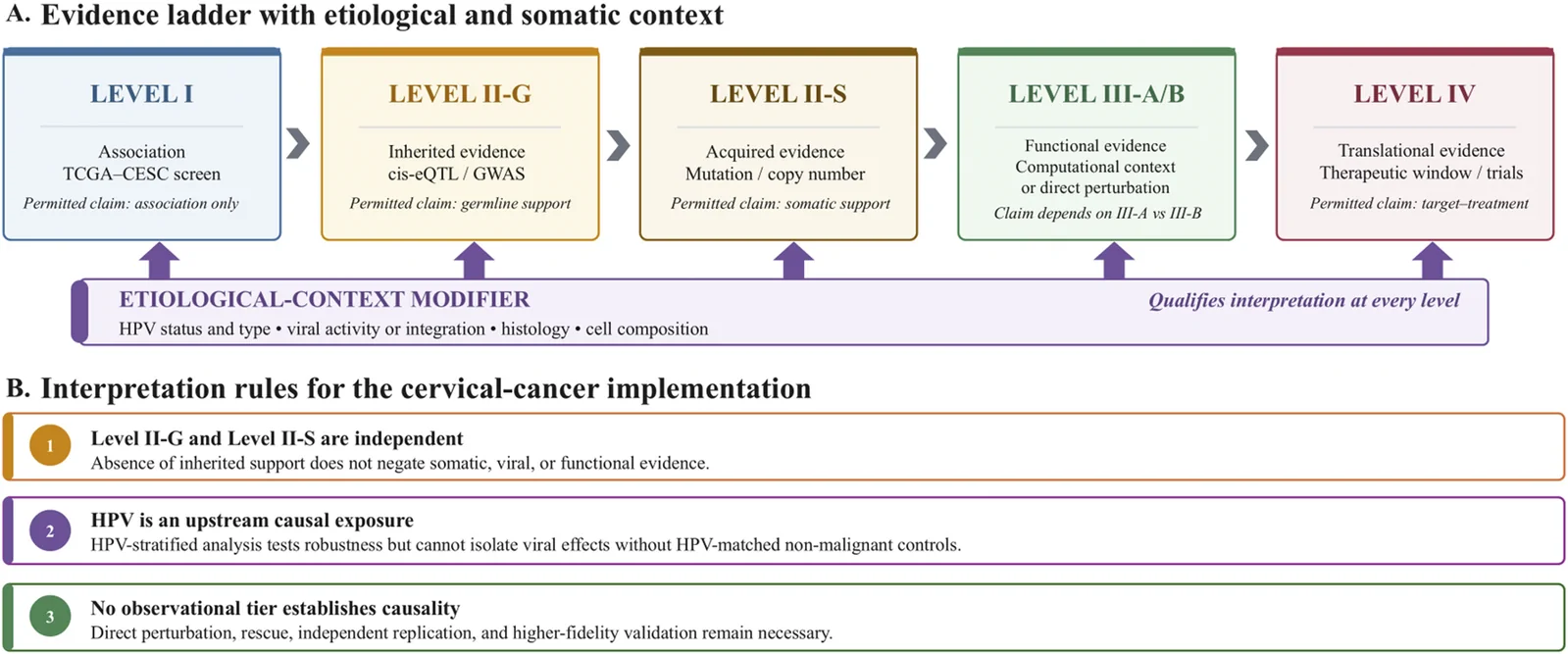
Cervical-cancer implementation of the Causal Integration Ladder (CIL). **(A)** CIL depicts the prospective genomic-discovery route from Level I association through Level II-G inherited and Level II-S somatic evidence, Level III-A computational context, Level III-B direct perturbation, and Level IV translational evidence. The horizontal arrows are not a compulsory historical sequence; the dashed clinical-entry arrow shows that established target-treatment relationships may enter at Level IV without imputed lower-level evidence. **(B)** Worked examples show Partial Level II-G for all four genes; CDC20 and KRT6A are Moderate Level III-A, whereas KRT5 and S100A9 are Context-only Level III-A. No exploratory candidate reaches Full Level II or Strong Level III-A; the indicated experiments are prospective.

The present work uses cervical cancer as the sole empirical application. The objectives are to (i) implement the CIL using a transparent public-data workflow; (ii) apply consistent decision rules across the manuscript, figures, and tables; (iii) evaluate a TCGA-CESC-derived candidate set without equating cross-trait signals with cervical causality; and (iv) specify cervical experiments that would most efficiently reduce uncertainty. Extension to other diseases is a conceptual scope statement, not a validation claim.

## 2 Materials and methods

### 2.1 Study design and public data resources

The study is a hypothesis-and-theory framework with a retrospective, public-data cervical-cancer implementation. Level I uses TCGA-CESC expression and clinical annotations. Level II integrates germline regulatory and association evidence. Level III-A integrates computational perturbation, pharmacogenomic, signature, and cell-compartment evidence. No new patient recruitment, wet-lab experiment, or clinical intervention was performed. Table 1 lists data versions and analytical roles.

**TABLE 1:** Public data resources used in the cervical-cancer CIL implementation.

Resource	Release/version	Role	Source
TCGA-CESC/GDC	GDC data release 40.0	Level I expression and clinical annotation	GDC portal; TCGA Research Network (2013)
NHGRI-EBI GWAS catalog	Associations downloaded April 2026	Contextual gene–trait annotation	Sollis et al. (2023)
GTEx portal	v8	cis-eQTL instruments	GTEx Consortium (2020)
Open targets platform	v25 GraphQL API	Precomputed QTL–GWAS colocalization	Ochoa et al. (2023)
PubMed	Searched through 19 July 2026	Cervical-cancer and genetic-evidence context	NCBI PubMed
DepMap	Public 26Q1	Chronos CRISPR dependency and model annotation	DepMap (2026); Arafeh et al. (2025)
DEMETER2	v6 combined	Orthogonal RNAi dependency	McFarland et al. (2018)
GDSC1/GDSC2	Fitted dose-response release 8.5	Expression–ln (IC50) correlation	Iorio et al. (2016)
SigCom LINCS	l1000_cp	Whole-signature reversal	Evangelista et al. (2022)
CZ CELLxGENE census	Where-my-gene v2	Single-cell compartment annotation	CZI Cell Science Program et al. (2025)

APIs, and releases are reported to make the evidence matrix auditable. A match returned by a database was not treated as causal evidence unless it met the relevant CIL, decision rule.

### 2.2 TCGA-CESC expression and candidate screen (level I)

STAR-normalized transcript-per-million (TPM) values and clinical annotations were retrieved for 306 TCGA-CESC tumours and 3 normal samples from GDC Data Release 40 (The Cancer Genome Atlas Research Network, 2013; 2017). Genes with median TPM <1 were excluded, and expression was transformed as log2 (TPM+1). Tumour–normal contrasts used a two-sample Welch t-test. Effect size, nominal p-value, and Benjamini–Hochberg false-discovery rate (FDR) were retained. Because the normal group contained only three samples, the 25-gene panel was defined as an exploratory effect-size-ranked screen (15 upregulated and 10 downregulated genes with |log2FC| ≥ 1.5 and nominal p < 0.05), not as a confirmatory FDR-significant set. FDR values are shown in Supplementary Table S1.

#### 2.2.1 HPV-stratified sensitivity analysis

HPV status and type were obtained from the published TCGA-CESC sample-level annotation (Panayiotou et al., 2020; The Cancer Genome Atlas Research Network, 2017) and matched by TCGA sample barcode. Of 291 annotated primary tumours, 280 were HPV-positive, 9 HPV-negative, and 2 indeterminates; indeterminate samples were excluded from group comparisons. Robustness was assessed by restricting the tumour component of each Level I effect-size estimate to HPV-positive cases and by exploratory Welch comparisons of log2 (RSEM+1) expression between HPV-positive and HPV-negative tumours. Benjamini–Hochberg correction was applied across the 25-gene panel. Because the HPV-negative group was small and enriched for non-squamous histologies, effect sizes and direction were emphasized, and HPV-specific effects were not claimed.

### 2.3 Clinical stratification

Samples were summarized descriptively by age group (<40, 40–60, and >60 years) and International Federation of Gynecology and Obstetrics (FIGO) stage. FIGO stages I–IV describe increasing anatomical extent: disease confined to the cervix (I), spread beyond the cervix without pelvic-wall/lower-vaginal involvement (II), pelvic-wall/lower-vaginal involvement or hydronephrosis (III), and adjacent-organ invasion or distant spread (IV). Because the archived analytical output did not retain group-level sample counts or patient-level test records, no age- or stage-association p-values are reported. Figure 2, Supplementary Figure S1 and Supplementary Table S5 show descriptive group means only.

**FIGURE 2:**
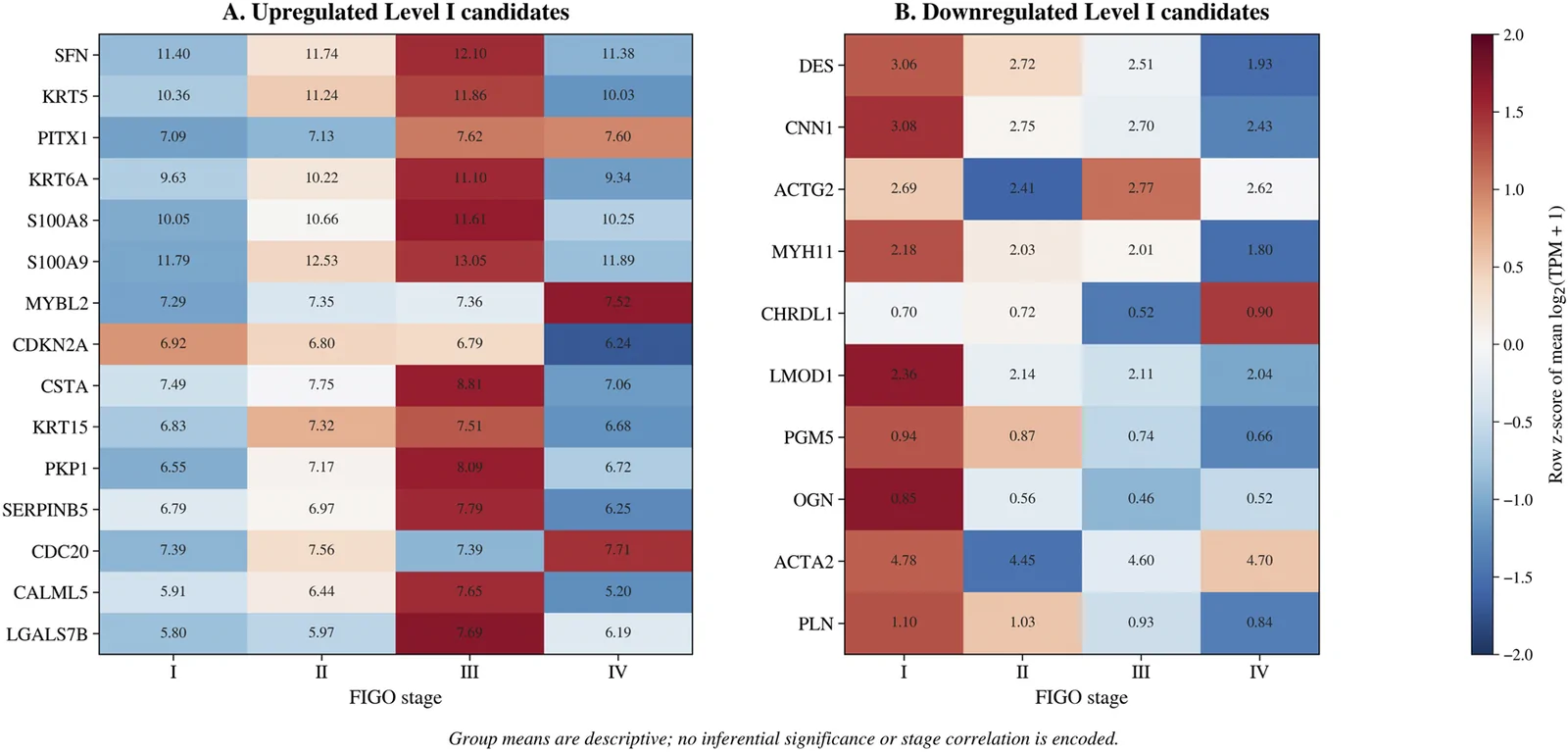
Descriptive expression patterns across FIGO stages for the 25 priority genes. Cells show mean log2(TPM+1) by stage; color represents row z-score. No inferential significance is encoded because group counts and patient-level test records were not retained in the archived analytical output.

### 2.4 Contextual GWAS annotation

Gene-linked associations were downloaded from the NHGRI-EBI GWAS Catalog in April 2026 (Sollis et al., 2023). The GWAS tier is a contextual annotation, not an independent causal tier. STRONG denotes multiple genome-wide signals with a disease-, immune-, or gene-linked molecular context judged relevant to the cervical analysis; MODERATE denotes a genome-wide signal with plausible biological context but limited specificity or replication; LIMITED denotes suggestive evidence or genome-wide associations confined to remote, unrelated traits; NONE denotes no retained association. Consequently, catalog p-value alone does not determine the colour tier. Cross-trait GWAS annotations cannot complete Level II. The harmonized gene-level genetic evidence and contextual GWAS annotations are reported in Supplementary Table S2 and S3 and visualized in Supplementary Figures S2-S4.

### 2.5 cis-eQTL and colocalization analysis (level II)

GTEx v8 cis-eQTL records were queried for each priority gene (The GTEx Consortium et al., 2020) as inherited regulatory evidence, designated Level II-G. Precomputed Open Targets QTL–GWAS colocalization records were screened (Ochoa et al., 2023), but records lacking complete study identifiers, traits, loci, alleles, and record-level provenance were excluded. Level II-G therefore concerns inherited susceptibility or regulation only. Its absence does not weaken evidence arising from acquired somatic alteration, HPV context, or functional perturbation.

### 2.6 Somatic mutation and copy-number analysis (level II-S)

TCGA Firehose Legacy whole-exome mutation calls, GISTIC 2.0 discrete copy-number calls, continuous Affymetrix SNP6 gene-level copy-number values, and RNA-seq V2 RSEM expression were analysed as Level II-S (The Cancer Genome Atlas Research Network, 2017). Mutation frequencies used 194 sequenced tumours as the denominator; silent, intronic, untranslated-region, intergenic, and flanking variants were excluded from the protein-altering count. Copy-number frequencies used 295 evaluable tumours. Spearman correlations between continuous copy number and log2 (RSEM+1) expression used matched samples and were corrected across the 25-gene panel. Shallow loss or single-copy gain was not equated with a focal driver alteration; interpretation required concordant expression association and retained separate reporting of high-level events.

#### 2.6.1 Eligibility assessment for genetic causal inference

Genetic causal estimation was considered but was ineligible for this implementation because the retained exposure and cervical-cancer outcome records did not provide aligned effect estimates for the same variant or a documented harmonized proxy. Allele-frequency substitution from another population cannot supply the missing variant-level coefficients, effect-allele alignment, or linkage-disequilibrium documentation. Accordingly, no Mendelian-randomization estimate or causal claim is reported. Any future analysis would require disease-matched summary statistics, harmonized cis-eQTL instruments, aligned alleles and effect estimates, linkage-disequilibrium handling, instrument-strength assessment, and appropriate sensitivity analyses.

### 2.7 Computational functional support (level III-A)

DepMap Public 26Q1 Chronos scores were screened across cervical-line models and pan-cancer models, and DEMETER2 v6 supplied orthogonal RNAi context (DepMap, 2026; Arafeh et al., 2025; McFarland et al., 2018). GDSC analyses follow the pharmacogenomic resource described by Iorio et al. (2016). GDSC1/2 release 8.5 expression–ln (IC50) Spearman correlations were treated as nominal exploratory associations; no multiplicity-adjusted drug claim is made. The retained export contained the most extreme cervical-line dependency value rather than a model-level distribution, so these values are presented as screening flags and cannot establish selectivity or Strong Level III-A. LINCS reversal is a whole-signature property (Evangelista et al., 2022), and CELLxGENE assignments are contextual compartment annotations (CZI Cell Science Program et al., 2025).

### 2.8 CIL decision rules and quality modifiers

CIL separates evidence completeness from confidence. Full Level II requires Level I association, a significant cis-eQTL, auditable cervical-cancer outcome colocalization at PP.H4 ≥ 0.80, and a valid harmonized causal estimate or equivalent independent genetic triangulation. Partial Level II-G denotes incomplete matched genetic evidence. Strong Level III-A requires reproducible cervical-line dependency across more than one relevant model plus an orthogonal support axis and model-level reporting; none of the present candidates meets that audit standard. Moderate Level III-A requires a preliminary cervical-line dependency signal, even when selectivity or model-level replication is unresolved. Context-only Level III-A denotes compartment annotation or nominal response association without a qualifying cervical dependency; it guides experiment selection but does not constitute functional support. Each label is accompanied by quality modifiers (Figure 3; Table 2).

**FIGURE 3:**
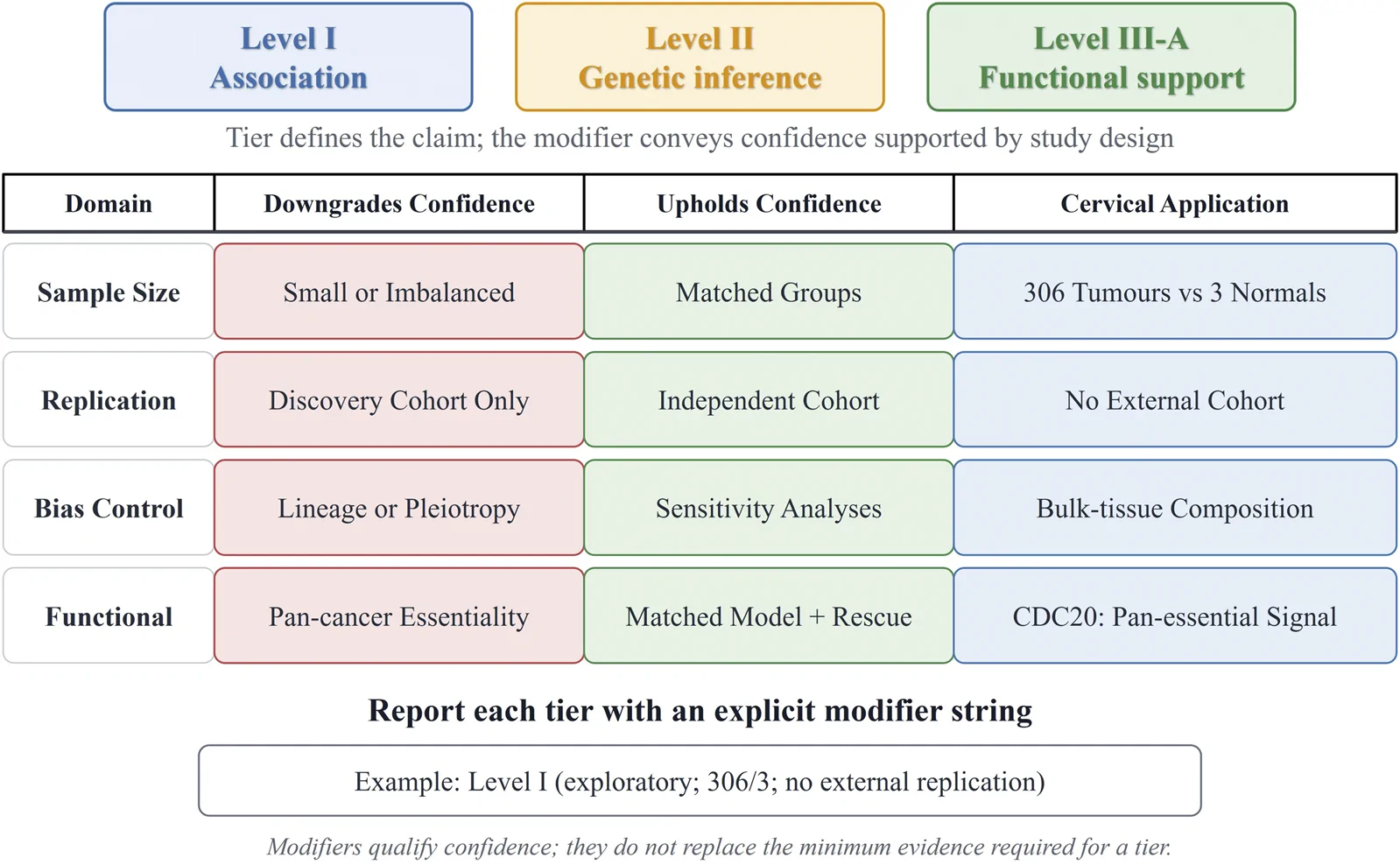
Quality-of-evidence modifiers within CIL tiers. A tier label is accompanied by study-level modifiers for sample size, design, replication, instrument strength, and bias-domain risk. These modifiers qualify confidence in the claim without substituting for the evidence required by the tier. In the cervical application, the Level I tumour–normal contrast is explicitly downgraded because only three normal samples were available.

**TABLE 2:** CIL definitions, classification rules, and principal quality modifiers.

Tier	Claim	Minimum operational evidence	Key modifiers
Level I	Disease association	Expression/clinical/multi-omics association; no causal claim	Small control group, no replication, lineage composition
Partial level II	Incomplete genetically constrained support	Documented cis-eQTL and/or relevant GWAS context, but incomplete matched genetic evidence	Source provenance, proxy tissue, ancestry/tissue match
Full level II	Complete genetically constrained support	Level I + auditable cervical-cancer colocalization (PP.H4 ≥ 0.80) + valid harmonized causal estimate or independent triangulation	Allele harmonization, pleiotropy, replication
Context-only level III-A	Context annotation	Nominal drug correlation, signature, or compartment annotation without qualifying cervical dependency	Multiplicity, tissue/cell origin, model relevance
Moderate level III-A	Preliminary dependency	Cervical-line dependency screening signal; selectivity or model-level replication unresolved	Model distribution, pan-essentiality, pan-cancer context
Strong level III-A	Convergent computational support	Reproducible dependency across ≥2 cervical models + orthogonal support + model-level reporting	Pan-essentiality, model representativeness
Level III-B	Direct experimental perturbation	Matched model, targeted perturbation, phenotype, and rescue/orthogonal validation	Off-target effects; controls
Level IV	Translational validation	Higher-fidelity efficacy, therapeutic window, and clinical validation	External validity, toxicity, endpoints

Full/Partial describes Level II, evidence completeness. Strong/Moderate describes Level III-A, functional-support strength. Neither modifier should be used as a universal numerical score.

### 2.9 Software and reproducibility

Analyses and document generation used Python 3.11 with pandas, NumPy, SciPy, matplotlib, requests, and python-docx. Supplementary Code 1 is a downstream cervical-cancer classification runner, not an end-to-end reproduction of upstream TCGA, GWAS, colocalization, dependency, pharmacogenomic, or single-cell analyses. It accepts a user-supplied differential-expression table, validates gene, log2FC, direction, and optional p/FDR fields, joins the locked and versioned Level II/III-A evidence tables, leaves unmatched genes at Level I, and produces a master classification table, summary figure, per-gene narrative, and run metadata. Reproduction of upstream evidence requires the separately cited source datasets and analysis-specific scripts. Adapting the runner to another disease requires newly curated and validated evidence tables rather than changing a disease label.

## 3 The causal integration ladder

### 3.1 Level I: associational architecture

Level I identifies disease-associated features from expression, clinical, genomic, epigenomic, proteomic, metabolomic, or imaging data. It is a nomination tier. Neither large effect size nor a very small p-value establishes a therapeutic mechanism. Quality modifiers record whether the association is replicated, adequately powered, corrected for multiple testing, and robust to cellular composition.

### 3.2 Level II: genetically constrained inference

At Level II, CIL evaluates whether inherited regulatory variation links a gene to a disease-relevant outcome. Users assess cis-eQTL support, disease-matched colocalization, and eligible causal estimates against the prespecified criteria and source-provenance requirements. A cis-eQTL alone shows that expression is genetically regulated; colocalization alone shows compatibility with a shared locus; and MR relies on assumptions about instrument validity. CIL assigns Full Level II only when evidence converges around a cervical-cancer outcome with complete source provenance. Reported HPV-susceptibility and cross-trait records lacking that audit trail were excluded from classification.

### 3.3 Level III-A, level III-B, and level IV

At Level III-A, CIL organizes scalable computational functional evidence. Dependency scores, drug-response correlations, perturbational signatures, and single-cell compartment annotations can inform experiment prioritization, but each evidence type remains model dependent. CIL assigns Level III-B only after direct, disease-relevant perturbation with appropriate controls and rescue or orthogonal confirmation. At Level IV, users evaluate whether the mechanism is supported by higher-fidelity models, therapeutic-window assessment, and clinical testing. This progression prevents the word ‘causal’ from being attached prematurely to a computational screen. Figure 1 arrows depict the prospective genomic-discovery route only. Clinically established target-treatment relationships may enter at Level IV and be mapped backward across domains without assuming that earlier levels were satisfied.

### 3.4 How CIL prioritizes the next experiment

Missing evidence may be more informative than the assigned classification. A Level I candidate needs matched genetic or independent expression replication; a Partial Level II-G candidate needs the absent matched colocalization or directional test; a Context-only or Moderate Level III-A candidate needs direct perturbation in a disease-relevant model; and a Strong Level III-A candidate needs rescue, non-malignant comparators, mechanism read-outs, and therapeutic-window assessment. The hierarchy therefore supports qualitative value-of-information logic without converting heterogeneous data into an opaque aggregate score.

## 4 Results: cervical-cancer implementation

### 4.1 Level I reveals epithelial, cell-cycle, inflammatory, and stromal programs

The exploratory TCGA-CESC contrast yielded the 15 genes with the largest positive log2FC values and the 10 genes with the largest negative log2FC values (Figures 4, 5, 6; Table 3; Supplementary Figures S2-S5). Upregulated genes included epithelial keratins (KRT5, KRT6A, KRT15), cell-cycle genes (SFN, MYBL2, CDKN2A, CDC20), and the S100A8/S100A9 inflammatory pair. Downregulated genes were dominated by smooth-muscle/stromal and cytoskeletal programs (DES, CNN1, ACTG2, MYH11, LMOD1, PGM5, and ACTA2). These patterns are biologically coherent but cannot distinguish tumour-cell programs from shifts in stromal or immune composition.

**FIGURE 4:**
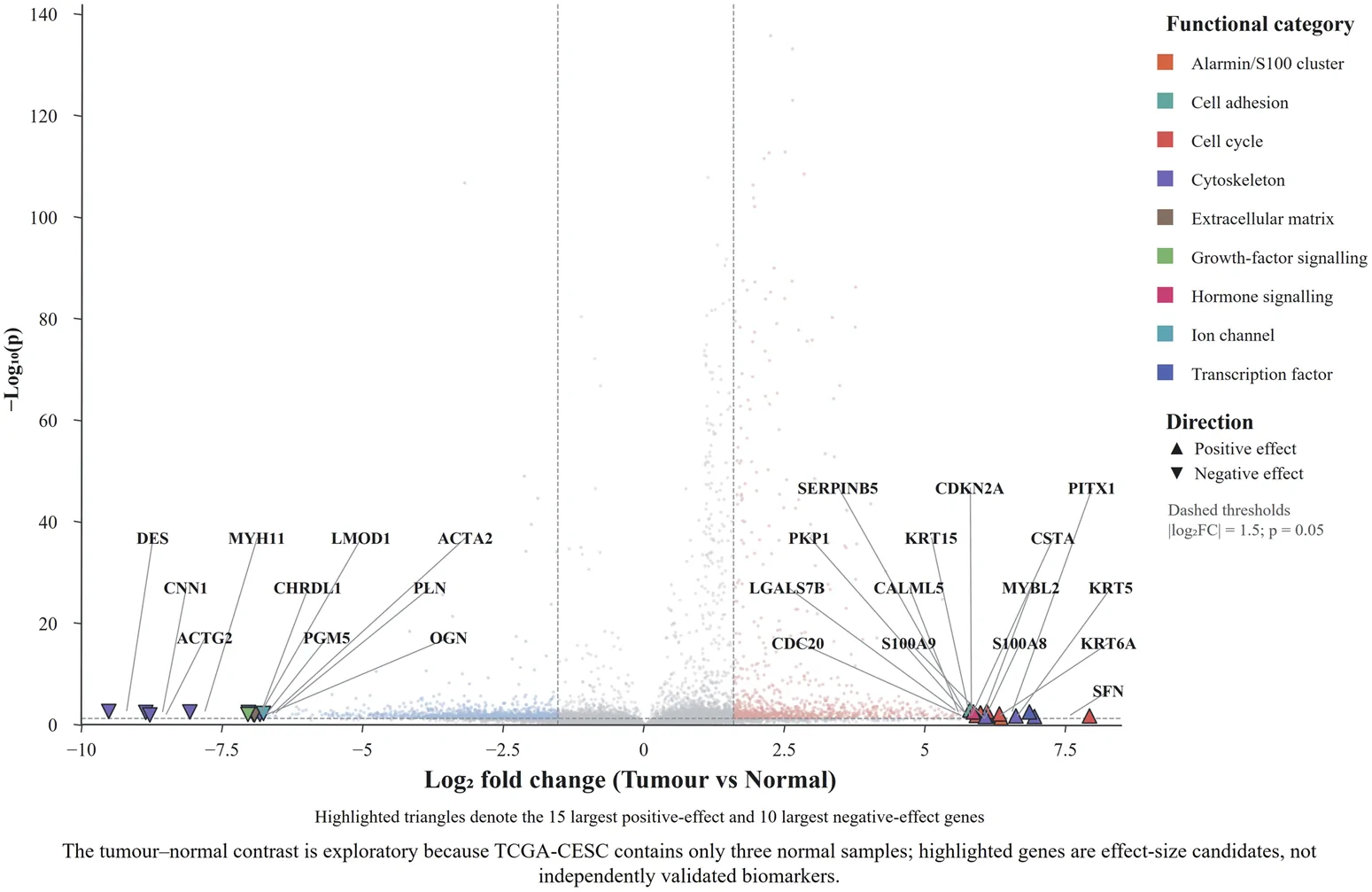
Exploratory tumour-versus-normal expression contrast in TCGA-CESC. The x-axis shows log2 fold change and the y-axis shows –log10(nominal p-value). Dashed vertical lines mark |log2FC| = 1.5; the horizontal line marks nominal p = 0.05. The 25 ranked priority genes are labelled. Because TCGA-CESC contains 306 tumours but only three normal samples, the figure is treated as a Level I candidate screen; false-discovery-rate values are reported in Supplementary Table S1 and no causal inference is made.

**FIGURE 5:**
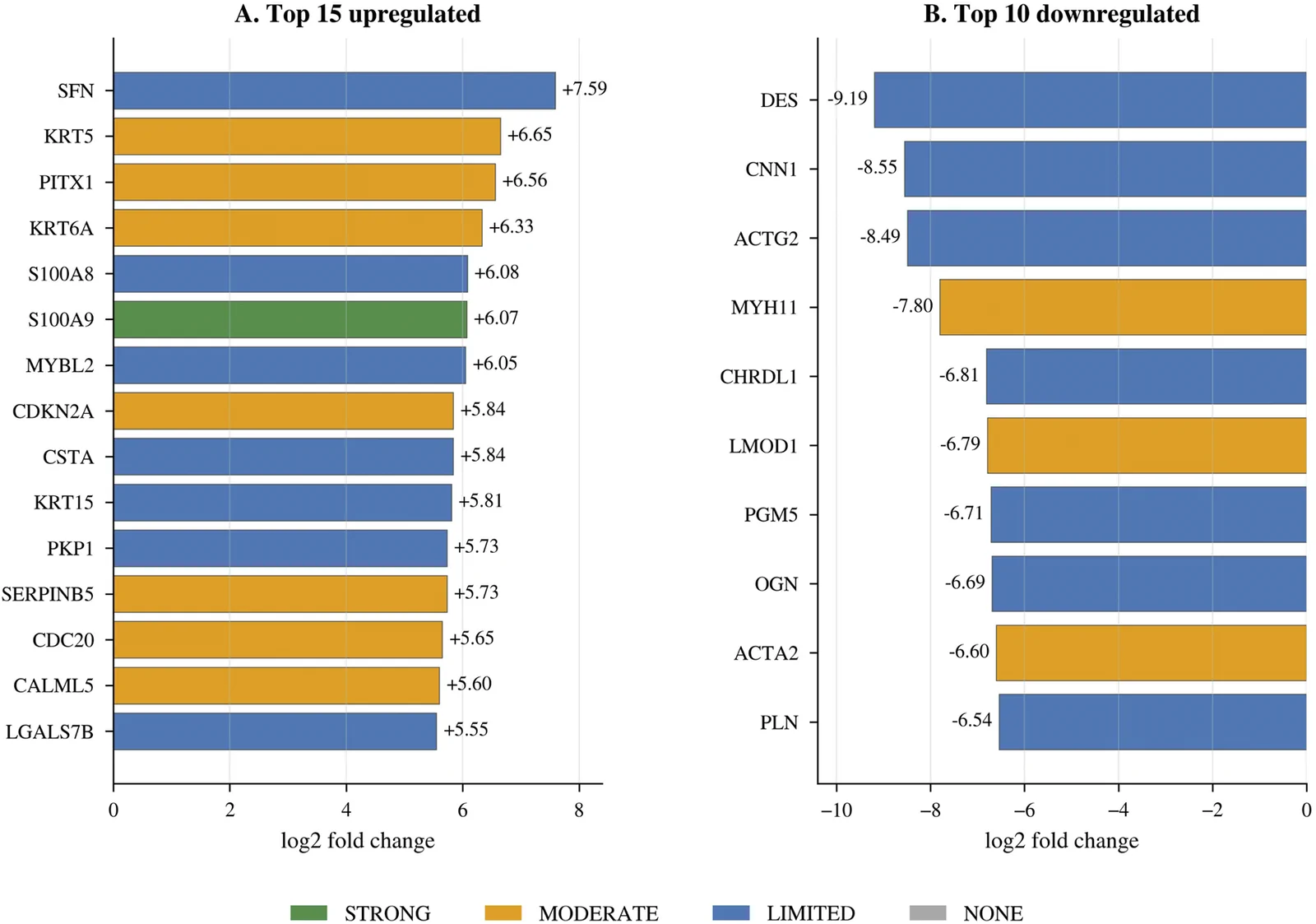
Ranked TCGA-CESC tumour–normal contrasts stratified by contextual GWAS annotation tier. **(A)** The 15 upregulated candidates. **(B)** The 10 downregulated candidates. Bars show log2 fold change; colors encode a relevance-weighted GWAS annotation (STRONG, MODERATE, LIMITED, or NONE). The tier is contextual and is not determined by p-value alone. Exact values are provided in Table 3 and Supplementary Table S1 and S3.

**FIGURE 6:**
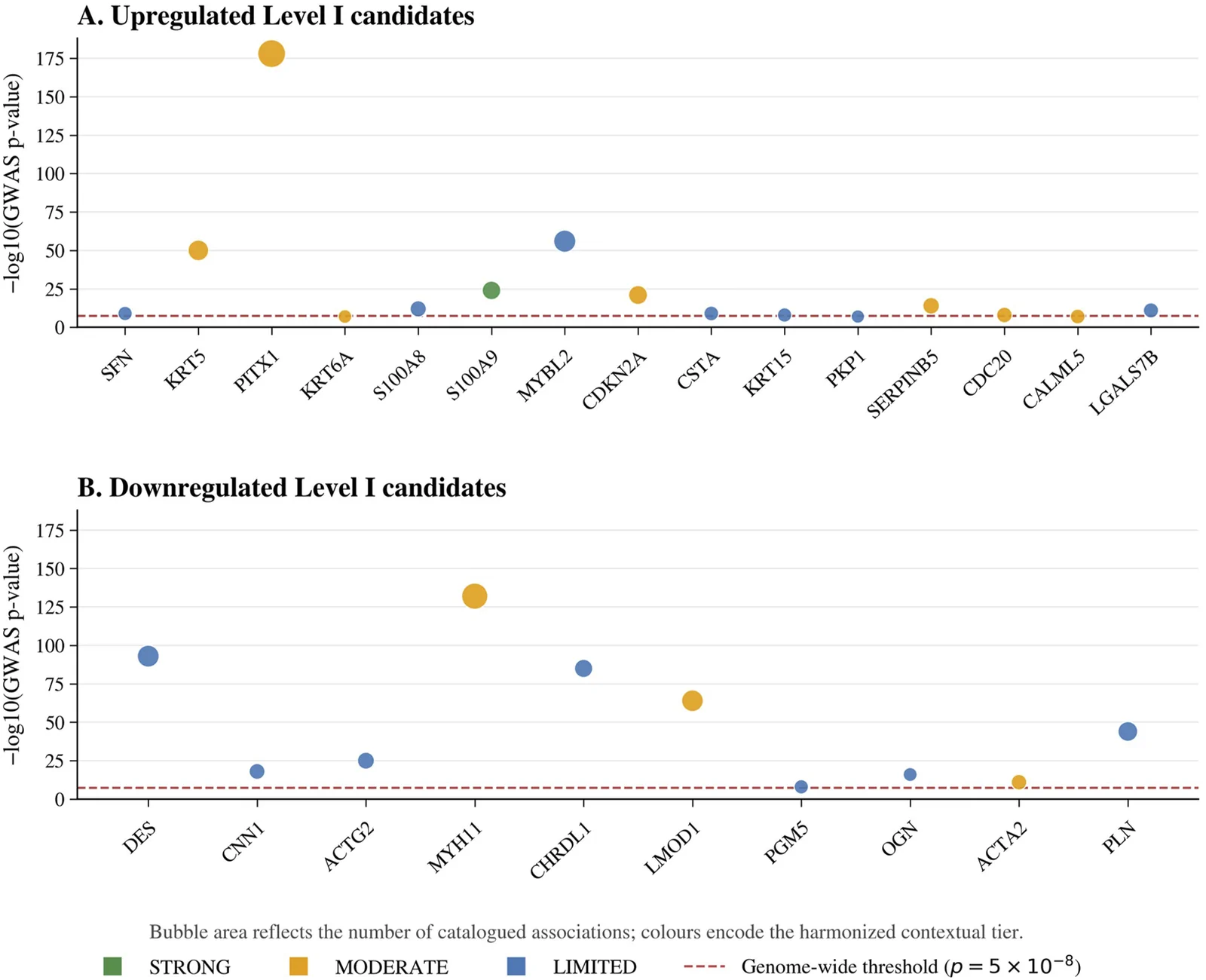
GWAS annotations for the 25 priority genes. Each bubble represents the most significant catalogued association linked to a gene; the y‐axis is ‐log10(p-value), bubble size reflects the number of catalogued associations, and color denotes the relevance-weighted annotation tier. The dashed line marks p = 5×10–8. Many associations concern traits other than cervical cancer and therefore provide context rather than Level II completion. Documented cis‐eQTL evidence is separated from reported colocalization records that lacked complete source‐level provenance and were therefore excluded. No candidate has auditable cervical‐cancer outcome colocalization plus a valid harmonized causal estimate; all remain Partial Level II‐G. Excluded PP.H4 values are not displayed. Exact source‐audit decisions are reported in Supplementary Tables S2 ‐S4. Reported best‐line Chronos values, nominal GDSC correlation counts, and cell‐compartment annotations are shown as exploratory context. Because model‐level dependency distributions and multiplicity‐adjusted drug results were not retained, no candidate is classified as Strong Level III‐A.

**TABLE 3:** TCGA-CESC 25-gene Level I panel and final CIL classification.

Gene	Direction	log2FC	Functional category	GWAS annotation	Final CIL label
DES	Down	−9.19	Cytoskeleton	LIMITED	Level I
CNN1	Down	−8.55	Cytoskeleton	LIMITED	Level I
ACTG2	Down	−8.49	Cytoskeleton	LIMITED	Level I
MYH11	Down	−7.80	Cytoskeleton	MODERATE	Level I
CHRDL1	Down	−6.81	Growth-factor signalling	LIMITED	Level I
LMOD1	Down	−6.79	Cytoskeleton	MODERATE	Level I
PGM5	Down	−6.71	Cytoskeleton	LIMITED	Level I
OGN	Down	−6.69	Extracellular matrix	LIMITED	Level I
ACTA2	Down	−6.60	Cytoskeleton	MODERATE	Level I
PLN	Down	−6.54	Ion channel	LIMITED	Level I
SFN	Up	+7.59	Cell cycle	LIMITED	Level I
KRT5	Up	+6.65	Cytoskeleton	MODERATE	Partial II + context-only III-A
PITX1	Up	+6.56	Transcription factor	MODERATE	Partial II + context-only III-A
KRT6A	Up	+6.33	Cytoskeleton	MODERATE	Partial II + moderate III-A
S100A8	Up	+6.08	Alarmin/S100 cluster	LIMITED	Partial II + context-only III-A
S100A9	Up	+6.07	Alarmin/S100 cluster	STRONG	Partial II + context-only III-A
MYBL2	Up	+6.05	Cell cycle	LIMITED	Level I
CDKN2A	Up	+5.84	Cell cycle	MODERATE	Partial II + context-only III-A
CSTA	Up	+5.84	Cell adhesion	LIMITED	Level I
KRT15	Up	+5.81	Cytoskeleton	LIMITED	Level I
PKP1	Up	+5.73	Cell adhesion	LIMITED	Level I
SERPINB5	Up	+5.73	Cell cycle	MODERATE	Partial II + context-only III-A
CDC20	Up	+5.65	Cell cycle	MODERATE	Partial II + moderate III-A
CALML5	Up	+5.60	Hormone signalling	MODERATE	Level I
LGALS7B	Up	+5.55	Cell adhesion	LIMITED	Level I

The Level I panel is an exploratory effect-size-ranked screen because only three normal samples were available. FDR, values are reported in Supplementary Table S1. The same canonical log2FC, and GWAS, tier values are used in all main and supplementary displays.

#### 4.1.1 HPV-stratified sensitivity analysis

HPV annotation was available for 291 primary tumours: 280 HPV-positive, 9 HPV-negative, and 2 indeterminates. All 25 Level I candidates retained their original direction after restricting the tumour estimate to HPV-positive cases (Supplementary Figure S6; Supplementary Table S7). Six genes differed between HPV-positive and HPV-negative tumours after panel-level FDR correction. Within the eight-gene audit set, CDKN2A, S100A9, and SERPINB5 were higher in HPV-positive tumours at FDR <0.05; KRT6A and S100A8 showed large but non-significant exploratory differences. CDC20 was comparatively stable. These comparisons do not isolate viral effects from histology or malignant transformation because only nine HPV-negative tumours and no HPV-matched non-malignant controls were available.

#### 4.1.2 Acquired somatic evidence

Somatic mutation was uncommon among the eight prioritized genes. In contrast, copy-number–expression associations were detected for CDKN2A, SERPINB5, PITX1, CDC20, S100A8, and S100A9 after correction across the 25-gene panel (Supplementary Figure S7; Supplementary Tables S8, S9). CDKN2A had one deep deletion, 58 shallow deletions, 52 gains, and one high-level amplification among 295 tumours, with a CNA–expression correlation of ρ = 0.372 (FDR = 6.5 × 10^−10^). Its expression was also higher in HPV-positive tumours, indicating that viral RB-pathway context and somatic dosage should be considered jointly. KRT5 and KRT6A showed no CNA–expression association, supporting interpretation as epithelial/HPV-context candidates rather than CNA-driven candidates.

Age- and FIGO-stage group means are shown descriptively (Figure 2; Supplementary Figure S1; Supplementary Table S5). They are not interpreted as patient-level associations because the retained output lacked group counts and reproducible patient-level test records.

### 4.2 Genetic evidence remains incomplete for all eight candidates

Figure 6 summarizes contextual GWAS annotations across the full 25-gene panel; these annotations do not constitute Level II evidence. Eight upregulated genes were carried forward for the Level II audit shown in Table 4 and Supplementary Tables S2-S4. Each had documented cis-eQTL and/or contextual GWAS information, but no candidate had a complete, source-auditable cervical-cancer colocalization record plus a valid harmonized causal estimate. All eight therefore remain Partial Level II-G. Unauditable PP.H4 values are neither reported nor displayed. Exact source-audit decisions are provided in Supplementary Tables S2-S4.

**TABLE 4:** Inherited Level II-G audit for eight TCGA-CESC priority genes.

Gene	cis-eQTL	Auditable cervical-cancer colocalization	MR eligible	Level II-G
KRT5	Yes	Incomplete	No	Partial II-G
KRT6A	Yes	Incomplete	No	Partial II-G
S100A9	Yes	Incomplete	Not evaluated	Partial II-G
S100A8	Yes	Incomplete	Not evaluated	Partial II-G
CDC20	Yes	Incomplete	Not evaluated	Partial II-G
CDKN2A	Yes	Incomplete	Not evaluated	Partial II-G
PITX1	Yes	Incomplete	Not evaluated	Partial II-G
SERPINB5	Yes	None retained	Not evaluated	Partial II-G

Documented cis-eQTL, evidence is separated from reported colocalization records that lacked complete source-level provenance and were therefore excluded. No candidate has auditable cervical-cancer outcome colocalization plus a valid harmonized causal estimate; all remain Partial Level II-G.

Cross-trait records noted in the working export were not used for promotion because complete study identifiers and record-level provenance were not retained. Thus, none of the eight candidates is presented as genetically established cervical-cancer causality.

### 4.3 Level III-A remains provisional after quality-modifier audit

CDC20 had a reported best cervical-line Chronos score of −2.74, but the model-level distribution was not retained and its pan-essentiality raises a selectivity concern. It is therefore classified as Moderate Level III-A and prioritized for therapeutic-window testing rather than labelled a selective dependency (Table 5; Supplementary Table S6). Context-only entries guide experiment selection but do not qualify as functional support; exact values and cautions are reported in Supplementary Table S6.

**TABLE 5:** Computational functional context for eight prioritized candidates.

Gene	CRISPR chronos	RNAi	GDSC nominal	Cell compartment	Level III-A
CDC20	−2.74*	No	n = 9	Cycling	Moderate
KRT6A	−0.55	No	n = 16	Epithelial	Moderate
CDKN2A	No	No	n = 9	Epithelial	Context-only
KRT5	No	No	n = 7	Epithelial	Context-only
PITX1	No	No	n = 8	Epithelial	Context-only
S100A8	No	No	n = 3	Myeloid	Context-only
S100A9	No	No	n = 17	Myeloid	Context-only
SERPINB5	No	No	n = 40	Epithelial	Context-only

Reported best-line Chronos values, nominal GDSC, correlation counts, and cell-compartment annotations are shown as exploratory context. Because model-level dependency distributions and multiplicity-adjusted drug results were not retained, no candidate is classified as Strong Level III-A.

KRT6A had a reported best cervical-line Chronos score of −0.55, but without model-level distribution or orthogonal RNAi support this remains a preliminary dependency flag. KRT6A is classified as Moderate Level III-A. The remaining six candidates are Context-only Level III-A because nominal drug correlations or compartment annotations do not satisfy the minimum dependency criterion. GDSC counts are nominal, LINCS reversal is panel-level, and S100A8/S100A9 localization supports microenvironmental rather than tumour-cell-autonomous hypotheses.

### 4.4 Cervical markers outside the expression-ranked panel

The Level I starting point determines which genes enter the upward workflow. CXCL8, IRF6, and the serum marker CA125/MUC16 were raised during review because they have cervical-cancer relevance but were not selected in the 25-gene effect-size-ranked panel. We did not assign new CIL tiers without a complete, harmonized evidence analysis. Instead, we use them to illustrate complementary entry routes. Experimental work links CXCL8 to cervical-carcinoma proliferation and apoptosis (Ding et al., 2021); IRF6 has tumour-suppressive evidence in cervical models (Ren et al., 2020); and elevated CA125 has prognostic associations in surgically treated cervical adenocarcinoma (Kim et al., 2021). Such candidates can enter CIL from a literature- or clinical-evidence starting point, but the provenance of that entry must be recorded and the same Level II/III criteria applied prospectively.

### 4.5 Clinical-anchor demonstration using established cervical-cancer therapies

To assess whether CIL can represent established therapeutic evidence without forcing every target through a germline-genetic route, we performed a *post hoc* clinical-anchor demonstration using three mechanistically distinct interventions supported by randomized cervical-cancer trials: VEGFA/VEGF-A inhibition with bevacizumab, PDCD1/PD-1 blockade with pembrolizumab, and F3/tissue-factor-directed delivery with tisotumab vedotin (Tewari et al., 2014; Colombo et al., 2021; Vergote et al., 2024). Each anchor is retained at Level IV for the specific target-treatment relationship (Table 6). Lower levels are reported as not assessed rather than inferred. This exercise also distinguishes therapeutic validation from disease causality: the clinical efficacy of an antiangiogenic agent, checkpoint inhibitor, or antibody-drug conjugate does not by itself establish that the engaged target initiated cervical carcinogenesis.

**TABLE 6:** Clinical-anchor demonstration of reverse entry into CIL.

Target/intervention	Cervical-cancer evidence	CIL representation	Interpretive boundary
VEGFA/VEGF-A; bevacizumab	Randomized phase III GOG-240: Survival benefit when bevacizumab was added to chemotherapy	Level IV for the anti-VEGF treatment relationship; levels I-II not assessed in this anchor	Supports therapeutic relevance; does not establish germline support or tumour-initiation causality
PDCD1/PD-1; pembrolizumab	Randomized phase III KEYNOTE-826: Benefit when pembrolizumab was added to chemotherapy, with or without bevacizumab	Level IV for PD-1 blockade in the trial-defined population; biomarker context recorded separately	Treatment efficacy is not equivalent to PDCD1 being a tumour-cell dependency or cervical-cancer initiating gene
F3/tissue factor; tisotumab vedotin	Randomized phase III innovaTV 301: Improved efficacy versus investigator-choice chemotherapy after progression	Level IV for tissue-factor-directed drug delivery; levels I-II not imputed	Validates a target-delivery relationship, not F3 as a causal cervical-cancer driver

All three anchors were representable without contradictory promotion: clinically demonstrated target-treatment relationships were preserved at Level IV, while absent genetic or tumour-initiating evidence remained unassigned. This is a coherence check rather than a performance benchmark; no sensitivity, specificity, calibration, or superiority claim is made.

## 5 Discussion

The cervical-cancer application shows why evidence types should not be collapsed into a single rank. All eight candidates have incomplete Level II evidence, and two Level III-A labels remain Moderate and six remain Context-only after applying reproducibility and reporting modifiers. The result is a prioritized experimental agenda rather than a list of validated targets.

This separation also prevents two recurrent overinterpretations. First, strong colocalization with an immune, anthropometric, or other-cancer trait is not cervical-cancer colocalization. Second, expression–drug-response correlations and LINCS signature reversal do not show that inhibiting the nominated gene will reproduce the drug effect. CIL retains those data as contextual support while restricting causal language to the evidence actually present.

For KRT6A, the most informative Level III-B study is inducible knockout or knockdown in several HPV-positive cervical models, followed by cDNA rescue, epithelial-state markers, proliferation, invasion, and comparison with non-malignant cervical keratinocytes. This would test whether the moderate Chronos dependency is reproducible and lineage specific. For CDC20, partial and time-controlled suppression with rescue should be paired with non-malignant cervical comparators and mitotic read-outs; the main question is therapeutic window, not whether complete loss impairs proliferation.

For S100A8/S100A9, tumour–myeloid co-culture, chemotaxis, cytokine blockade, and spatial localization are more informative than tumour-cell viability assays because the single-cell evidence is myeloid. For CDKN2A, perturbation should be stratified by RB-pathway state and evaluated with CDK4/6-inhibitor response. KRT5 requires a mechanism-focused study of HPV entry/persistence or epithelial state rather than assuming that absence of dependency negates its Level II evidence. These experiments map directly to the missing or conflicting evidence identified by CIL.

HPV is an environmental infectious exposure and an etiological prerequisite, so host-genetic association with HPV susceptibility is not equivalent to a gene causing cervical cancer. Future availability of harmonized cervical-cancer outcome and molecular-QTL summary statistics may permit formal MR with appropriate sensitivity analyses. Such analyses could strengthen genetic triangulation but would not independently establish a molecular mechanism or equate susceptibility to HPV infection with malignant progression. Anthro-demographic, clinicopathological, and biochemical factors may also structure cervical-cancer risk and observed molecular associations, reinforcing the need for population-aware validation (Kushwah et al., 2022). Bulk RNA-seq also mixes malignant, immune, stromal, and vascular compartments. The CELLxGENE annotation is a coarse contextual check, not a deconvolution of TCGA samples.

The most important design limitation is the use of only three TCGA normal samples. Welch testing does not compensate for such a small control group, and several top effect-size candidates do not meet FDR <0.05. Accordingly, Level I is explicitly exploratory and requires independent, adequately sampled cervical tissue validation. Other limitations include proxy-tissue eQTLs, precomputed rather than primary-summary-statistic colocalization, the absence of harmonized cervical-cancer MR inputs, pan-cancer pharmacogenomic correlations, incomplete representation of cervical histological subtypes, and no direct perturbation of the eight exploratory candidates. The three-target clinical-anchor demonstration is a *post hoc* representational check, not an externally adjudicated validation set. Its small size and clinically selected composition preclude estimates of sensitivity, specificity, calibration, recovery, or comparative ranking performance.

Portability depends on disease architecture and cannot be achieved by applying an unchanged cervical-cancer pipeline. Virus-associated cancers require viral status, activity, integration, and latency programs as etiological-context modifiers; EBV-related lymphomas, for example, would require EBV latency and cell-of-origin annotation. Cancers arising from strong inherited predisposition require pathogenic germline variants, allele-specific loss, and second-hit events. Environment-associated cancers require exposure measurement and mutational-signature evidence. In cancers without a dominant upstream exposure, greater weight must be placed on convergent somatic, longitudinal, spatial, and perturbational evidence. Cohort size, control selection, histological composition, and assay completeness determine which claims are portable.

CIL is designed for gene- or mechanism-level therapeutic hypotheses. It should not force composite or treatment-selection biomarkers, such as tumour mutation burden, microsatellite instability, or a serum protein measurement, into a gene tier. The ladder depicts the prospective discovery route from association to translation; it is not a compulsory historical sequence and lower-domain evidence is not imputed when a target enters from clinical practice. A reverse-entry workflow can begin with a clinically used intervention, identify the directly engaged gene or mechanism, and annotate each evidence domain independently. Level IV therefore records intervention-specific translational evidence, not proof that the target is a disease-initiating driver or that Levels I-II have been satisfied.

A machine-readable CIL implementation could support carefully bounded artificial-intelligence workflows. Structured evidence records, explicit provenance, and fixed decision rules could enable retrieval, source checking, gap detection, and consistency auditing across large evidence collections. For example, the reference tables and runner could be exposed as resources and tools through a Model Context Protocol (MCP)-compatible service, allowing an AI client to query evidence and propose the next analysis expected to reduce uncertainty (Model Context Protocol, 2026). Such a system should not autonomously promote candidates: source qualification, disease-context judgment, and final tier assignment require expert review, logged provenance, and reproducible outputs. This is a prospective implementation pathway, not a capability evaluated in the present study.

The logic of CIL may be transferable, but only cervical cancer is empirically examined in this study. In a non-cancer application, tumour–normal expression might be replaced by a matched-tissue case–control contrast or another disease-relevant Level I phenotype. Level II outcomes, tissues, confounders, and Level III models must then be redefined and validated for that disease. DepMap and GDSC are cancer resources and cannot simply be declared disease agnostic. The supplied runner is therefore intentionally cervical specific; broader generalizability remains a testable hypothesis for future work.

## 6 Conclusion

The Causal Integration Ladder provides an auditable way to move from cervical-cancer genomic association to therapeutic-target hypotheses without erasing uncertainty. The revised implementation separates inherited Level II-G evidence from acquired Level II-S evidence and treats HPV as an etiological-context modifier. HPV-positive restriction preserved the direction of all 25 Level I candidates, while somatic analysis identified gene-specific CNA–expression associations but little recurrent mutation. These results refine candidate interpretation without establishing causality. Direct perturbation, rescue, independent replication, therapeutic-window analysis, and higher-fidelity validation remain necessary before causal or translational claims are made.

## Data Availability

All analysed data were obtained from the public resources and releases listed in Table 1. Derived evidence tables are provided in the Supplementary Material. The cervical-cancer CIL classification runner, reference files, example input, expected outputs, and instructions are available at https://github.com/Dr-SS03/tcga-cesc-gwas-analysis. The runner integrates curated evidence tables; it does not recreate upstream TCGA, GTEx, Open Targets, DepMap, GDSC, LINCS, or CELLxGENE analyses. The repository does not redistribute controlled-access or third-party source datasets.

## References

[B1] Arafeh R. Shibue T. Dempster J. M. Hahn W. C. Vazquez F. (2025). The present and future of the cancer dependency map. Nat. Rev. Cancer 25, 59–73. 10.1038/s41568-024-00763-x 39468210

[B2] Behan F. M. Iorio F. Picco G. Gonçalves E. Beaver C. M. Migliardi G. (2019). Prioritization of cancer therapeutic targets using CRISPR-Cas9 screens. Nature 568, 511–516. 10.1038/s41586-019-1103-9 30971826

[B3] Colombo N. Dubot C. Lorusso D. Caceres M. V. Hasegawa K. Shapira-Frommer R. (2021). Pembrolizumab for persistent, recurrent, or metastatic cervical cancer. N. Engl. J. Med. 385, 1856–1867. 10.1056/NEJMoa2112435 34534429

[B4] CZI Cell Science Program, Abdulla S. Aevermann B. Assis P. Badajoz S. Bell S. M. (2025). CZ CELLxGENE discover: a single-cell data platform for scalable exploration, analysis and modeling of aggregated data. Nucleic Acids Res. 53, D886–D900. 10.1093/nar/gkae1142 39607691 PMC11701654

[B5] Davey Smith G. Hemani G. (2014). Mendelian randomization: genetic anchors for causal inference in epidemiological studies. Hum. Mol. Genet. 23, R89–R98. 10.1093/hmg/ddu328 25064373 PMC4170722

[B6] DepMap (2026). DepMap portal. DepMap portal. Available online at: https://depmap.org/portal/data_page/?tab=allData (Accessed May 4, 2026).

[B7] Ding H.-M. Zhang H. Wang J. Zhou J.-H. Shen F.-R. Ji R.-N. (2021). miR-302c-3p and miR-520a-3p suppress the proliferation of cervical carcinoma cells by targeting CXCL8. Mol. Med. Rep. 23, 322. 10.3892/mmr.2021.11961 33760117 PMC7974325

[B8] Evangelista J. E. Clarke D. J. B. Xie Z. Lachmann A. Jeon M. Chen K. (2022). SigCom LINCS: data and metadata search engine for a million gene expression signatures. Nucleic Acids Res. 50, W697–W709. 10.1093/nar/gkac328 35524556 PMC9252724

[B9] Giambartolomei C. Vukcevic D. Schadt E. E. Franke L. Hingorani A. D. Wallace C. (2014). Bayesian test for colocalisation between pairs of genetic association studies using summary statistics. PLoS Genet. 10, e1004383. 10.1371/journal.pgen.1004383 24830394 PMC4022491

[B10] Gupta M. K. Kushwah A. S. Singh R. Srivastava K. Banerjee M. (2023). Genetic and epigenetic alterations in MGMT gene and correlation with concomitant chemoradiotherapy (CRT) in cervical cancer. J. Cancer Res. Clin. Oncol. 149, 15159–15170. 10.1007/s00432-023-05305-w PMC11797194 37634205

[B11] Iorio F. Knijnenburg T. A. Vis D. J. Bignell G. R. Menden M. P. Schubert M. (2016). A landscape of pharmacogenomic interactions in cancer. Cell. 166, 740–754. 10.1016/j.cell.2016.06.017 27397505 PMC4967469

[B12] Kim N. Park W. Cho W. K. Bae D. S. Kim B. G. Lee J. W. (2021). Significance of serum CA125 level in surgically resected cervical adenocarcinoma with adverse features. J. Gynecol. Oncol. 32, e72. 10.3802/jgo.2021.32.e72 34132070 PMC8362813

[B13] Kushwah A. S. Mishra R. Srivastava K. Mishra S. Banerjee M. (2022). Anthro-demographic, clinico-pathological and biochemical risk factors in cervical cancer. Int. J. Health Sci. 6 (S8), 3641–3653. 10.53730/ijhs.v6nS8.12025

[B14] Kushwah A. S. Srivastava K. Mishra R. Banerjee M. (2024). Impact of human papillomavirus (HPV) infection on the treatment outcome of concomitant chemoradiotherapy (CRT) in cervical cancer. CCTR 20, 320–326. 10.2174/1573394719666230807161948

[B15] McFarland J. M. Ho Z. V. Kugener G. Dempster J. M. Montgomery P. G. Bryan J. G. (2018). Improved estimation of cancer dependencies from large-scale RNAi screens using model-based normalization and data integration. Nat. Commun. 9, 4610. 10.1038/s41467-018-06916-5 30389920 PMC6214982

[B16] McLaughlin-Drubin M. E. Münger K. (2009). Oncogenic activities of human papillomaviruses. Virus Res. 143, 195–208. 10.1016/j.virusres.2009.06.008 19540281 PMC2730997

[B17] Model Context Protocol (2026). Model context protocol specification, version 2026-07-28. Available online at: https://modelcontextprotocol.io/specification/2026-07-28 (Accessed August 11, 2026).

[B18] Moody C. A. Laimins L. A. (2010). Human papillomavirus oncoproteins: pathways to transformation. Nat. Rev. Cancer 10, 550–560. 10.1038/nrc2886 20592731

[B19] Ochoa D. Hercules A. Carmona M. Suveges D. Baker J. Malangone C. (2023). The next-generation open targets platform: reimagined, redesigned, rebuilt. Nucleic Acids Res. 51, D1353–D1359. 10.1093/nar/gkac1046 36399499 PMC9825572

[B20] Panayiotou T. Michael S. Zaravinos A. Demirag E. Achilleos C. Strati K. (2020). Human papillomavirus E7 binds Oct4 and regulates its activity in HPV-associated cervical cancers. PLoS Pathog. 16, e1008468. 10.1371/journal.ppat.1008468 32298395 PMC7228134

[B21] Pingault J.-B. O’Reilly P. F. Schoeler T. Ploubidis G. B. Rijsdijk F. Dudbridge F. (2018). Using genetic data to strengthen causal inference in observational research. Nat. Rev. Genet. 19, 566–580. 10.1038/s41576-018-0020-3 29872216

[B22] Porcu E. Sjaarda J. Lepik K. Carmeli C. Darrous L. Sulc J. (2021). Causal inference methods to integrate omics and complex traits. Cold Spring Harb. Perspect. Med. 11, a040493. 10.1101/cshperspect.a040493 32816877 PMC8091955

[B23] Ren Y. Dong J. He P. Liang Y. Wu L. Wang J. (2020). miR-587 promotes cervical cancer by repressing interferon regulatory factor 6. J. Gene Med. 22, e3257. 10.1002/jgm.3257 32749750

[B24] Richmond R. C. Hemani G. Tilling K. Davey Smith G. Relton C. L. (2016). Challenges and novel approaches for investigating molecular mediation. Hum. Mol. Genet. 25, R149–R156. 10.1093/hmg/ddw197 27439390 PMC5036871

[B25] Singh S. Nema R. Banerjee M. Kushwah A. S. (2025). Genetic and epigenetic landscape of O6-methylguanine-DNA methyltransferase (MGMT): implications for DNA repair and cancer therapeutics. Explor Target Antitumor Ther. 6, 1002335. 10.37349/etat.2025.1002335 40895460 PMC12394067

[B26] Sollis E. Mosaku A. Abid A. Buniello A. Cerezo M. Gil L. (2023). The NHGRI-EBI GWAS catalog: knowledgebase and deposition resource. Nucleic Acids Res. 51, D977–D985. 10.1093/nar/gkac1010 36350656 PMC9825413

[B27] Tewari K. S. Sill M. W. Long H. J. Penson R. T. Huang H. Ramondetta L. M. (2014). Improved survival with bevacizumab in advanced cervical cancer. N. Engl. J. Med. 370, 734–743. 10.1056/NEJMoa1309748 24552320 PMC4010094

[B28] The Cancer Genome Atlas Research Network, Weinstein J. N. Collisson E. A. Mills G. B. Shaw K. R. M. Ozenberger B. A. (2013). The cancer genome atlas pan-cancer analysis project. Nat. Genet. 45, 1113–1120. 10.1038/ng.2764 PMC3919969 24071849

[B29] The Cancer Genome Atlas Research Network (2017). Albert einstein college of medicine analytical biological services barretos cancer hospital baylor college of medicine beckman research institute of city of hope integrated genomic and molecular characterization of cervical cancer. Nature 543, 378–384. 10.1038/nature21386 28112728 PMC5354998

[B30] The GTEx Consortium, Aguet F. Anand S. Ardlie K. G. Gabriel S. Getz G. A. (2020). The GTEx consortium atlas of genetic regulatory effects across human tissues. Science 369, 1318–1330. 10.1126/science.aaz1776 32913098 PMC7737656

[B31] Tsherniak A. Vazquez F. Montgomery P. G. Weir B. A. Kryukov G. Cowley G. S. (2017). Defining a cancer dependency map. Cell. 170, 564–576.e16. 10.1016/j.cell.2017.06.010 28753430 PMC5667678

[B32] Vergote I. González-Martín A. Fujiwara K. Kalbacher E. Cibula D. Mirza M. R. (2024). Tisotumab vedotin as second- or third-line therapy for recurrent cervical cancer. N. Engl. J. Med. 391, 44–55. 10.1056/NEJMoa2313811 38959480

