# Supplementary material for: The causal integration ladder: a multilevel evidence framework for therapeutic target evaluation in cervical cancer
Source: Front Syst Biol. 2026 Sep 18;6:1874355. doi: 10.3389/fsysb.2026.1874355 (PMC13630610; doi:10.3389/fsysb.2026.1874355)
Supplement: Supplementary file 1 [file DataSheet1.docx]

**SUPPLEMENTARY MATERIAL**

**The Causal Integration Ladder: A Multilevel Evidence Framework for Therapeutic Target Evaluation in Cervical Cancer**

# Supplementary Figures

**Figure S1: Descriptive age-group expression of the 25-gene priority panel. Cells show mean log2(TPM+1); colour represents row z-score. Age groups were <40, 40–60, and >60 years. No inferential significance is encoded because group counts and patient-level test records were not retained in the archived analytical output.**

**Figure S2: Level I effect sizes stratified by the harmonized contextual GWAS annotation tier. The log2FC and tier values are reported in Table 3 and Supplementary Tables S1 and S3.**

**Figure S3: Cervical-cancer Level I context. Left, sentinel-variant lollipop plot ranked by the canonical minimum catalogued GWAS p-value; colour denotes the harmonized contextual GWAS tier and the dashed line marks p = 5×10−8. Right, Level I effect size versus nominal expression p-value, with colour indicating functional category and marker direction indicating tumour up- or downregulation. The GWAS associations include non-cervical traits and do not independently establish Level II.**

**Figure S4: Gene-by-functional-category map. Marker colour represents the contextual GWAS tier, shape represents expression direction, and marker size represents catalogued GWAS significance. The figure is descriptive and does not encode causal evidence.**

**Figure S5: Integrated overview of the cervical-cancer Level I landscape. Left, the full tumour–normal volcano distribution with the 25-gene panel labelled; neutral background points were darkened without changing their coordinates. Right, distribution of the 25 genes across functional categories, stratified by contextual GWAS support.**

# Supplementary Tables

**Supplementary Table S1. Exploratory TCGA-CESC tumour–normal expression contrasts for the 25-gene panel.**

| **Gene** | **log2FC** | **p-value** | **FDR** | **Direction** |
| --- | --- | --- | --- | --- |
| DES | -9.19 | 2.31 × 10⁻⁶ | 0.028 | Down |
| CNN1 | -8.55 | 4.12 × 10⁻⁶ | 0.037 | Down |
| ACTG2 | -8.49 | 8.63 × 10⁻⁶ | 0.075 | Down |
| MYH11 | -7.80 | 3.28 × 10⁻⁶ | 0.030 | Down |
| CHRDL1 | -6.81 | 7.71 × 10⁻⁶ | 0.065 | Down |
| LMOD1 | -6.79 | 4.35 × 10⁻⁶ | 0.038 | Down |
| PGM5 | -6.71 | 8.10 × 10⁻⁶ | 0.072 | Down |
| OGN | -6.69 | 8.94 × 10⁻⁶ | 0.078 | Down |
| ACTA2 | -6.60 | 7.18 × 10⁻⁶ | 0.065 | Down |
| PLN | -6.54 | 5.94 × 10⁻⁶ | 0.054 | Down |
| SFN | +7.59 | 1.01 × 10⁻⁵ | 0.097 | Up |
| KRT5 | +6.65 | 1.26 × 10⁻⁵ | 0.118 | Up |
| PITX1 | +6.56 | 3.61 × 10⁻⁶ | 0.032 | Up |
| KRT6A | +6.33 | 1.13 × 10⁻⁵ | 0.106 | Up |
| S100A8 | +6.08 | 1.67 × 10⁻⁵ | 0.163 | Up |
| S100A9 | +6.07 | 1.68 × 10⁻⁵ | 0.163 | Up |
| MYBL2 | +6.05 | 6.82 × 10⁻⁶ | 0.061 | Up |
| CDKN2A | +5.84 | 3.22 × 10⁻⁶ | 0.030 | Up |
| CSTA | +5.84 | 1.07 × 10⁻⁵ | 0.101 | Up |
| KRT15 | +5.81 | 1.15 × 10⁻⁵ | 0.108 | Up |
| PKP1 | +5.73 | 5.81 × 10⁻⁶ | 0.054 | Up |
| SERPINB5 | +5.73 | 4.89 × 10⁻⁶ | 0.046 | Up |
| CDC20 | +5.65 | 9.31 × 10⁻⁶ | 0.085 | Up |
| CALML5 | +5.60 | 3.55 × 10⁻⁶ | 0.031 | Up |
| LGALS7B | +5.55 | 1.84 × 10⁻⁶ | 0.016 | Up |

The panel was selected by effect size and nominal p < 0.05 because only three normal samples were available. FDR is reported transparently; rows with FDR ≥ 0.05 are not described as confirmatory differential expression.

**Supplementary Table S2. Source-audit status of Level II evidence for eight prioritized genes.**

| **Gene** | **Documented cis-eQTL** | **Tissue** | **Colocalization record** | **Audit decision** | **Level II** |
| --- | --- | --- | --- | --- | --- |
| KRT5 | rs688861; p=3.6×10−20 | Esophagus mucosa | Incomplete source record | Excluded; MR not eligible | Partial II |
| KRT6A | rs11439938; p=4.3×10−7 | Whole blood | Incomplete source record | Excluded; MR not eligible | Partial II |
| S100A9 | rs3014874; p=2.6×10−13 | Whole blood | Incomplete source record | Excluded | Partial II |
| S100A8 | rs3014874; p=1.2×10−8 | Whole blood | Incomplete source record | Excluded | Partial II |
| CDC20 | rs1199039; p=3.7×10−6 | Esophagus mucosa | Incomplete source record | Excluded | Partial II |
| CDKN2A | rs3731198; p=2.8×10−7 | Whole blood | Incomplete source record | Excluded | Partial II |
| PITX1 | rs6596201; p=1.2×10−21 | Whole blood | Incomplete source record | Excluded | Partial II |
| SERPINB5 | rs6567350; p=5.0×10−16 | Esophagus mucosa | None retained | Not applicable | Partial II |

Full Level II requires source-auditable cervical-cancer outcome colocalization and a valid harmonized causal estimate or equivalent independent triangulation. Reported PP.H4 values lacking complete study identifiers, trait labels, locus/allele information, and record-level provenance were excluded.

**Supplementary Table S3. GWAS annotations for the 25-gene panel.**

| **Gene** | **Lead SNP** | **Locus** | **Top GWAS Trait** | **Min GWAS p-value** | **GWAS Associations (n)** | **Evidence Tier** |
| --- | --- | --- | --- | --- | --- | --- |
| DES | rs907683 | 2:219434819 | Heart rate | 9.0×10⁻⁹⁴ | 103 | LIMITED |
| CNN1 | rs8100751 | 19:11549197 | ACP5 protein levels | 3.0×10⁻¹⁸ | 8 | LIMITED |
| ACTG2 | rs1050146 | 2:73901306 | Height | 1.0×10⁻²⁵ | 14 | LIMITED |
| MYH11 | rs3915425 | 16:15818687 | Heart rate | 7.0×10⁻¹³³ | 247 | MODERATE |
| CHRDL1 | rs112683038 | X:110666798 | LDL phospholipids | 6.0×10⁻⁸⁶ | 28 | LIMITED |
| LMOD1 | rs2820312 | 1:201900129 | Body mass index | 3.0×10⁻⁶⁴ | 97 | MODERATE |
| PGM5 | rs11142461 | 9:68444973 | EPCAM protein levels | 1.0×10⁻⁸ | 2 | LIMITED |
| OGN | rs777898257 | 9:92393819 | OGN protein levels | 2.0×10⁻¹⁶ | 1 | LIMITED |
| ACTA2 | rs12761227 | 10:88988837 | FAS protein levels | 5.0×10⁻¹² | 6 | MODERATE |
| PLN | rs11153730 | 6:118346359 | QT interval | 1.0×10⁻⁴⁴ | 63 | LIMITED |
| SFN | rs144171499 | 1:26869648 | SHBG levels | 7.0×10⁻¹⁰ | 4 | LIMITED |
| KRT5 | rs11170164 | 12:52519884 | Basal cell carcinoma | 1.0×10⁻⁵⁰ | 78 | MODERATE |
| PITX1 | rs3805663 | 5:135030510 | Height | 4.0×10⁻¹⁷⁸ | 315 | MODERATE |
| KRT6A | rs576050939 | 12:52502619 | GLIPR1 protein levels | 5.0×10⁻⁸ | 3 | MODERATE |
| S100A8 | rs3795391 | 1:153390629 | Monocyte count | 2.0×10⁻¹² | 17 | LIMITED |
| S100A9 | rs3014874 | 1:153365467 | S100A11/S100A12 ratio | 1.0×10⁻²⁴ | 39 | STRONG |
| MYBL2 | rs285206 | 20:43667902 | Height | 8.0×10⁻⁵⁷ | 106 | LIMITED |
| CDKN2A | rs7874405 | 9:21980945 | Platelet count | 2.0×10⁻²¹ | 44 | MODERATE |
| CSTA | rs4678176 | 3:122290328 | Calcium levels | 4.0×10⁻¹⁰ | 7 | LIMITED |
| KRT15 | rs200702391 | 17:41515873 | Varicose veins | 3.0×10⁻⁹ | 5 | LIMITED |
| PKP1 | rs575701911 | 1:201266078 | GLIPR1 protein levels | 7.0×10⁻⁸ | 2 | LIMITED |
| SERPINB5 | rs12454742 | 18:63489373 | Serpin B5 levels | 2.0×10⁻¹⁴ | 11 | MODERATE |
| CDC20 | rs945508 | 1:156937289 | Subcortical volume | 8.0×10⁻⁹ | 9 | MODERATE |
| CALML5 | rs200038048 | 10:5471087 | Brainstem volume | 4.0×10⁻⁸ | 3 | MODERATE |
| LGALS7B | rs140695578 | 19:38774589 | LGALS4 protein levels | 9.0×10⁻¹² | 4 | LIMITED |

The annotation tier is relevance weighted; it is not a recording of minimum p-value or association count. Many top traits are not cervical-cancer outcomes and cannot complete Level II.

**Supplementary Table S4. Genetic causal-estimation eligibility note.**

| **Gene** | **Reported exposure variant(s)** | **Reported outcome variant** | **Harmonized same variant/proxy** | **Eligibility problem** | **Decision** |
| --- | --- | --- | --- | --- | --- |
| KRT5 | rs688861 / rs599466 reported across records | rs1707792 | No | Different variants; effect alleles and LD proxy relationship unavailable | Causal estimate not eligible |
| KRT6A | rs11439938 / rs644054 reported across records | rs1707792 | No | Different variants; effect alleles and LD proxy relationship unavailable | Causal estimate not eligible |

No genetic causal estimate was calculated because the retained exposure and cervical-cancer outcome records lacked aligned coefficients for the same variant or a documented harmonized proxy. Allele-frequency substitution cannot repair missing effect estimates, effect-allele alignment, or linkage-disequilibrium documentation. This table records an exclusion decision, not an MR analysis.

**Supplementary Table S5. Descriptive age- and FIGO-stage group means.**

| **Gene** | **Age <40** | **Age 40–60** | **Age >60** | **FIGO I** | **FIGO II** | **FIGO III** | **FIGO IV** |
| --- | --- | --- | --- | --- | --- | --- | --- |
| SFN | 11.67 | 11.53 | 11.69 | 11.40 | 11.74 | 12.10 | 11.38 |
| KRT5 | 11.08 | 10.48 | 11.35 | 10.36 | 11.24 | 11.86 | 10.03 |
| PITX1 | 7.29 | 7.04 | 7.65 | 7.09 | 7.13 | 7.62 | 7.60 |
| KRT6A | 10.19 | 9.85 | 10.17 | 9.63 | 10.22 | 11.10 | 9.34 |
| S100A8 | 10.78 | 10.17 | 10.88 | 10.05 | 10.66 | 11.61 | 10.25 |
| S100A9 | 12.40 | 11.99 | 12.42 | 11.79 | 12.53 | 13.05 | 11.89 |
| MYBL2 | 7.17 | 7.43 | 7.36 | 7.29 | 7.35 | 7.36 | 7.52 |
| CDKN2A | 7.04 | 6.78 | 6.67 | 6.92 | 6.80 | 6.79 | 6.24 |
| CSTA | 7.76 | 7.60 | 8.24 | 7.49 | 7.75 | 8.81 | 7.06 |
| KRT15 | 6.68 | 6.82 | 8.30 | 6.83 | 7.32 | 7.51 | 6.68 |
| PKP1 | 7.06 | 6.73 | 7.56 | 6.55 | 7.17 | 8.09 | 6.72 |
| SERPINB5 | 7.19 | 6.83 | 7.05 | 6.79 | 6.97 | 7.79 | 6.25 |
| CDC20 | 7.43 | 7.53 | 7.30 | 7.39 | 7.56 | 7.39 | 7.71 |
| CALML5 | 5.45 | 6.45 | 6.96 | 5.91 | 6.44 | 7.65 | 5.20 |
| LGALS7B | 5.47 | 6.28 | 6.97 | 5.80 | 5.97 | 7.69 | 6.19 |
| DES | 2.56 | 3.05 | 2.57 | 3.06 | 2.72 | 2.51 | 1.93 |
| CNN1 | 2.61 | 3.12 | 2.71 | 3.08 | 2.75 | 2.70 | 2.43 |
| ACTG2 | 2.19 | 2.87 | 2.63 | 2.69 | 2.41 | 2.77 | 2.62 |
| MYH11 | 1.79 | 2.26 | 2.07 | 2.18 | 2.03 | 2.01 | 1.80 |
| CHRDL1 | 0.56 | 0.72 | 0.76 | 0.70 | 0.72 | 0.52 | 0.90 |
| LMOD1 | 2.09 | 2.40 | 2.03 | 2.36 | 2.14 | 2.11 | 2.04 |
| PGM5 | 0.67 | 0.97 | 0.85 | 0.94 | 0.87 | 0.74 | 0.66 |
| OGN | 0.88 | 0.67 | 0.49 | 0.85 | 0.56 | 0.46 | 0.52 |
| ACTA2 | 4.47 | 4.82 | 4.52 | 4.78 | 4.45 | 4.60 | 4.70 |
| PLN | 0.75 | 1.19 | 1.04 | 1.10 | 1.03 | 0.93 | 0.84 |

Values are mean log2(TPM+1). No p-values or patient-level association claims are reported because group counts and patient-level test records were not retained.

**Supplementary Table S6. Audited Level III-A computational context.**

| **Gene** | **Level II** | **CRISPR** | **RNAi** | **GDSC correlations** | **Compartment** | **Level III-A** | **Caution** |
| --- | --- | --- | --- | --- | --- | --- | --- |
| CDC20 | Partial II | −2.74; strong/pan-essential | −0.26; no | 9; cisplatin | Progenitor/cycling | Moderate III-A | Therapeutic-window concern; best-line/nominal evidence only |
| KRT6A | Partial II | −0.55; dependency | +0.01; no | 16; oxaliplatin | Stratified epithelial | Moderate III-A | Context-specific; best-line/nominal evidence only |
| CDKN2A | Partial II | −0.08; no | +0.16; no | 9; palbociclib | Secretory epithelial | Context-only III-A | Exploratory drug correlation; best-line/nominal evidence only |
| KRT5 | Partial II | −0.21; no | +0.04; no | 7; oxaliplatin | Squamous/epithelial | Context-only III-A | No cervical dependency; best-line/nominal evidence only |
| PITX1 | Partial II | −0.33; no | −0.46; no | 8; GSK1059615 | Squamous epithelial | Context-only III-A | No cervical dependency; best-line/nominal evidence only |
| S100A8 | Partial II | −0.19; no | −0.11; no | 3; alpelisib | Myeloid/inflammatory | Context-only III-A | Microenvironmental hypothesis; best-line/nominal evidence only |
| S100A9 | Partial II | −0.27; no | −0.08; no | 17; palbociclib | Myeloid/inflammatory | Context-only III-A | Microenvironmental hypothesis; best-line/nominal evidence only |
| SERPINB5 | Partial II | −0.15; no | −0.30; no | 40; oxaliplatin | Squamous/epithelial | Context-only III-A | Exploratory drug correlation; best-line/nominal evidence only |

GDSC values are counts of nominal correlations and are not multiplicity-adjusted drug-efficacy evidence. Chronos values are reported best-line screening values; model-level distributions were not retained. Under the prespecified minimum criterion, CDC20 and KRT6A are Moderate Level III-A because they retain a cervical-line dependency screening signal; the other six candidates are Context-only Level III-A because drug correlations or compartment annotations alone do not qualify as functional support. No candidate meets Strong Level III-A criteria.

**Figure S6. HPV-positive restriction preserves the direction of the 25 Level I effect-size candidates. Red points denote the eight prioritized genes. The sensitivity estimates update the tumour component while retaining the original normal reference; it is not an independent differential-expression validation.**

**Figure S7. Continuous copy-number–expression associations for the eight prioritized genes. Blue bars indicate FDR < 0.05 after correction across the 25-gene panel. Association does not by itself establish focal driver status.**

**Supplementary Table S7. HPV-stratified expression sensitivity results for the 25 Level I candidates.**

| **Gene** | **Direction** | **Original log2FC** | **HPV+ sensitivity log2FC** | **HPV+ minus HPV−** | **P** | **FDR** | **Direction retained** |
| --- | --- | --- | --- | --- | --- | --- | --- |
| DES | Down | -9.19 | -9.23 | -1.16 | 0.227 | 0.258 | Yes |
| CNN1 | Down | -8.55 | -8.59 | -1.25 | 0.052 | 0.0876 | Yes |
| ACTG2 | Down | -8.49 | -8.54 | -1.73 | 0.015 | 0.0541 | Yes |
| MYH11 | Down | -7.80 | -7.84 | -1.35 | 0.054 | 0.0876 | Yes |
| CHRDL1 | Down | -6.81 | -6.84 | -0.98 | 0.403 | 0.403 | Yes |
| LMOD1 | Down | -6.79 | -6.83 | -1.32 | 0.027 | 0.0613 | Yes |
| PGM5 | Down | -6.71 | -6.77 | -2.04 | 0.092 | 0.128 | Yes |
| OGN | Down | -6.69 | -6.72 | -0.84 | 0.287 | 0.311 | Yes |
| ACTA2 | Down | -6.60 | -6.65 | -1.47 | 0.008 | 0.0427 | Yes |
| PLN | Down | -6.54 | -6.57 | -0.93 | 0.154 | 0.183 | Yes |
| LGALS7B | Up | 5.55 | 5.67 | 4.00 | 0.009 | 0.0427 | Yes |
| CALML5 | Up | 5.60 | 5.72 | 3.93 | 0.024 | 0.0597 | Yes |
| CDC20 | Up | 5.65 | 5.66 | 0.30 | 0.318 | 0.331 | Yes |
| PKP1 | Up | 5.73 | 5.83 | 3.19 | 0.051 | 0.0876 | Yes |
| SERPINB5 | Up | 5.73 | 5.89 | 5.22 | 0.003 | 0.0346 | Yes |
| KRT15 | Up | 5.81 | 5.95 | 4.61 | 0.022 | 0.0597 | Yes |
| CDKN2A | Up | 5.84 | 5.99 | 4.71 | 0.0001 | 0.0152 | Yes |
| CSTA | Up | 5.84 | 5.98 | 4.43 | 0.0083 | 0.0427 | Yes |
| MYBL2 | Up | 6.05 | 6.07 | 0.55 | 0.0983 | 0.129 | Yes |
| S100A9 | Up | 6.07 | 6.19 | 3.83 | 0.0103 | 0.0427 | Yes |
| S100A8 | Up | 6.08 | 6.19 | 3.54 | 0.0352 | 0.0732 | Yes |
| KRT6A | Up | 6.33 | 6.48 | 4.78 | 0.0205 | 0.0597 | Yes |
| PITX1 | Up | 6.56 | 6.62 | 1.89 | 0.114 | 0.143 | Yes |
| KRT5 | Up | 6.65 | 6.76 | 3.58 | 0.0722 | 0.106 | Yes |
| SFN | Up | 7.59 | 7.66 | 2.39 | 0.0561 | 0.0876 | Yes |

**Supplementary Table S8. Integrated HPV and somatic results for the eight prioritized genes.**

| **Gene** | **HPV+ minus HPV−** | **HPV FDR** | **Protein-altering mutation** | **Any CNA** | **CNA-expression rho** | **CNA FDR** |
| --- | --- | --- | --- | --- | --- | --- |
| CDC20 | 0.30 | 0.331 | 2/194 | 36.6% | 0.232 | 0.000241 |
| CDKN2A | 4.71 | 0.0152 | 3/194 | 38.0% | 0.372 | 6.51e-10 |
| KRT5 | 3.58 | 0.106 | 2/194 | 22.7% | 0.009 | 0.935 |
| KRT6A | 4.78 | 0.0597 | 2/194 | 22.7% | -0.017 | 0.879 |
| PITX1 | 1.89 | 0.143 | 1/194 | 32.2% | 0.271 | 1.28e-05 |
| S100A8 | 3.54 | 0.0732 | 0/194 | 55.9% | 0.197 | 0.00196 |
| S100A9 | 3.83 | 0.0427 | 1/194 | 55.9% | 0.192 | 0.00249 |
| SERPINB5 | 5.22 | 0.0346 | 1/194 | 38.6% | 0.339 | 1.65e-08 |

**Supplementary Table S9. Somatic mutation and copy-number results for the complete 25-gene Level I panel.**

| **Gene** | **Protein-altering mutation** | **−2** | **−1** | **+1** | **+2** | **CNA-expression rho** | **FDR** |
| --- | --- | --- | --- | --- | --- | --- | --- |
| ACTA2 | 0/194 | 6 | 82 | 16 | 1 | -0.051 | 0.598 |
| ACTG2 | 1/194 | 0 | 16 | 68 | 0 | -0.046 | 0.601 |
| CALML5 | 0/194 | 0 | 64 | 27 | 1 | 0.047 | 0.601 |
| CDC20 | 2/194 | 0 | 13 | 92 | 3 | 0.232 | 0.000241 |
| CDKN2A | 3/194 | 1 | 58 | 52 | 1 | 0.372 | 6.51e-10 |
| CHRDL1 | 1/194 | 0 | 59 | 48 | 1 | 0.008 | 0.935 |
| CNN1 | 0/194 | 3 | 75 | 35 | 5 | 0.091 | 0.218 |
| CSTA | 0/194 | 0 | 9 | 165 | 16 | 0.342 | 1.63e-08 |
| DES | 0/194 | 8 | 109 | 13 | 1 | 0.123 | 0.0733 |
| KRT15 | 3/194 | 3 | 35 | 46 | 1 | -0.001 | 0.989 |
| KRT5 | 2/194 | 0 | 15 | 51 | 1 | 0.009 | 0.935 |
| KRT6A | 2/194 | 0 | 15 | 51 | 1 | -0.017 | 0.879 |
| LGALS7B | 1/194 | 0 | 27 | 80 | 12 | 0.065 | 0.447 |
| LMOD1 | 2/194 | 0 | 7 | 148 | 2 | 0.040 | 0.655 |
| MYBL2 | 2/194 | 0 | 16 | 112 | 5 | 0.529 | 5.05e-21 |
| MYH11 | 3/194 | 0 | 31 | 51 | 4 | 0.031 | 0.747 |
| OGN | 2/194 | 1 | 55 | 52 | 0 | -0.120 | 0.0771 |
| PGM5 | 3/194 | 2 | 53 | 53 | 1 | -0.127 | 0.0672 |
| PITX1 | 1/194 | 1 | 63 | 30 | 1 | 0.271 | 1.28e-05 |
| PKP1 | 1/194 | 0 | 7 | 148 | 2 | 0.231 | 0.000241 |
| PLN | 0/194 | 3 | 79 | 25 | 2 | 0.028 | 0.754 |
| S100A8 | 0/194 | 0 | 7 | 148 | 10 | 0.197 | 0.00196 |
| S100A9 | 1/194 | 0 | 7 | 148 | 10 | 0.192 | 0.00249 |
| SERPINB5 | 1/194 | 1 | 79 | 33 | 1 | 0.339 | 1.65e-08 |
| SFN | 1/194 | 2 | 31 | 78 | 1 | 0.208 | 0.00111 |
